# The Effects of Combined Exposure to Bisphenols and Perfluoroalkyls on Human Perinatal Stem Cells and the Potential Implications for Health Outcomes

**DOI:** 10.3390/ijms241915018

**Published:** 2023-10-09

**Authors:** Andrea Di Credico, Giulia Gaggi, Ines Bucci, Barbara Ghinassi, Angela Di Baldassarre

**Affiliations:** 1 Reprogramming and Cell Differentiation Lab, Center for Advanced Studies and Technology (CAST), 66100 Chieti, Italy; andrea.dicredico@unich.it (A.D.C.); ines.bucci@unich.it (I.B.); b.ghinassi@unich.it (B.G.); angela.dibaldassarre@unich.it (A.D.B.); 2 Department of Medicine and Aging Sciences, “G. D’Annunzio” University of Chieti-Pescara, 66100 Chieti, Italy; 3 UdA TechLab Center (UdATech), 66100 Chieti, Italy

**Keywords:** Bisphenol A (BPA), Bisphenol S (BPS), perfluorooctane sulfonate (PFOS), perfluorooctanoic acid (PFOA), endocrine disruptors, perinatal stem cells, plastic pollutants, fetal membrane mesenchymal stromal cells (hFM-MSCs), amniotic fluid stem cells (hAFSCs)

## Abstract

The present study investigates the impact of two endocrine disruptors, namely Bisphenols (BPs) and Perfluoroalkyls (PFs), on human stem cells. These chemicals leach from plastic, and when ingested through contaminated food and water, they interfere with endogenous hormone signaling, causing various diseases. While the ability of BPs and PFs to cross the placental barrier and accumulate in fetal serum has been documented, the exact consequences for human development require further elucidation. The present research work explored the effects of combined exposure to BPs (BPA or BPS) and PFs (PFOS and PFOA) on human placenta (fetal membrane mesenchymal stromal cells, hFM-MSCs) and amniotic fluid (hAFSCs)-derived stem cells. The effects of the xenobiotics were assessed by analyzing cell proliferation, mitochondrial functionality, and the expression of genes involved in pluripotency and epigenetic regulation, which are crucial for early human development. Our findings demonstrate that antenatal exposure to BPs and/or PFs may alter the biological characteristics of perinatal stem cells and fetal epigenome, with potential implications for health outcomes at birth and in adulthood. Further research is necessary to comprehend the full extent of these effects and their long-term consequences.

## 1. Introduction

There is increasing concern about the health-threatening role of Bisphenols (BPs) and Perfluoroalkyls (PFs), two classes of chemical pollutants that are abundantly used in industrial applications to produce plastics, food packaging, and common household items [1]. These compounds can leach from plastics, contaminating the environment, food, and water, thus entering the human body, mainly through ingestion [2]. It has been shown that BPs and PFs act as endocrine disruptors (EDs), mimicking the action of natural steroid hormones [3] and leading to a wide spectrum of diseases [4,5,6]. Most of the studies about EDs are conducted by analyzing the effects of a single compound and do not take into account the possible interactions among the different chemicals. This generates a gap between the results from these studies and the real exposure effects because humans and animals are simultaneously exposed to a combination of different EDs (cocktail effect) that, when combined, may have additive or synergic activity.

The success of pregnancy and fetal growth is also dependent on placentation. This process begins soon after blastocyst implantation to form a mature placenta, which supports embryo development. Steroid hormones play a pivotal role in controlling placental formation and functionality. Due to the extensive use of plastic derivatives in human life, particular attention should be paid to antenatal exposure to chemicals leached from daily objects that may interfere with the in utero endocrine landscape. Early phases of human development are very susceptible to chemical toxicity and hormonal perturbation, which often result in poor outcomes at birth and later in life [7,8]. Recent data revealed that BPs, such as Bisphenol A (BPA) and S (BPS), and PFs, such as perfluorooctane sulfonate (PFOS) and perfluorooctanoic acid (PFOA), cross the placental barrier, accumulating in the placenta, fetal serum, and amniotic fluid [9,10,11,12]. It has been reported that the dysregulation of estradiol increases the risk of developing preeclampsia in pregnant women [13], and high BPA levels in the mother’s serum impact both placental morphology [12] and birth weight [14]. However, although both epidemiological and experimental studies have associated BPs and PFs exposure with detrimental health effects, their toxicity remains debated, mainly because epidemiological studies are useful in identifying associations and not causal links. Moreover, experimental studies frequently employ high exposure doses of EDs (above the actual daily exposure of humans), and a combination of EDs is not applied [15].

Developmental toxicity studies are usually conducted in animal models, but there is a high necessity for advancing more appropriate approaches that mimic in vitro human development. Due to the ethical restrictions in the use of human embryonic stem cells, new cellular models should be used to collect preliminary information on the developmental toxicity of these xenobiotics. Human fetal annexes, including the placenta and amniotic fluid, represent a valuable source of stem cells for research applications. In particular, fetal membrane mesenchymal stromal cells (hFM-MSCs) and amniotic fluid stem cells (hAFSCs) are isolated from the amniochorionic membrane of the placenta and amniotic fluid, respectively. These cells have intermediate characteristics between pluripotent (which can differentiate into cells belonging to all three germ layers) and multipotent cells (which can differentiate in one or more cell types belonging to only one germ layer), but they are non-tumorigenic and free from ethical concerns [16,17,18,19,20]. Given that stem cells constitute intrinsic components of embryonic development, investigating the impact of EDs on stem cells assumes paramount importance in elucidating the health implications associated with prenatal exposure to these environmental contaminants. In particular, since hFM-MSCs are isolated from the amniochorionic membrane of the placenta, they allow us to study the effects of EDs on the “environment” where the embryo develops. Conversely, the hAFSCs is a cell population that derives from both the amnioblasts and embryo, giving a representation of the conceptus status. Thus, this approach represents an appropriate model to clarify the potential developmental toxicity of in utero ED exposure.

The present study aimed to investigate the effects of antenatal exposure to different classes of EDs (BPA, BPS, PFOS, and PFOA), both as a single compound and in combination, on cellular models that mimic the in utero milieu. We used two cell types to recapitulate such a situation: hFM-MSCs and hAFSCs. The first ones derive from the placenta and represent the “environment” in which the embryo develops, while hAFSCs originate mainly from the respiratory, intestinal, and urinary tracts, giving a representation of the embryo. The effects of the selected EDs have been tested on these two cell types by analyzing cell proliferation by impedance and Ki67 expression, mitochondrial functionality, and the expression of genes involved in pluripotency and epigenetic regulation, which play a pivotal role soon after conception and in the first stages of human development.

## 2. Results

The potential developmental toxicity of BPs and PFs has been evaluated by treating the hFM-MSCs and hAFSCs with 0.1 μM of BPA, BPS, PFOS, and PFOA since such a concentration is within the range of BP and PF levels detected in the maternal and infant samples. Cells were exposed to a single chemical or to a cocktail in which EDs were paired into the following combinations: BPA + PFOS, BPA + PFOA, BPS + PFOS, BPS + PFOA, and BPA + BPS + PFOS + PFOA (ALL). The effects of the exposure on cell proliferation, mitochondrial health, and pluripotency and epigenetic regulator genes were then evaluated.

### 2.1. BPs and PFs Effects on hFM-MSCs Biology

#### 2.1.1. Proliferation and Cytotoxicity

**Figure 1:**
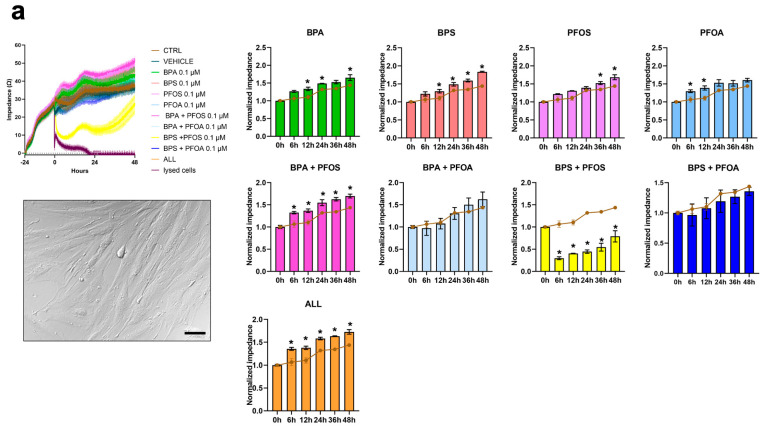

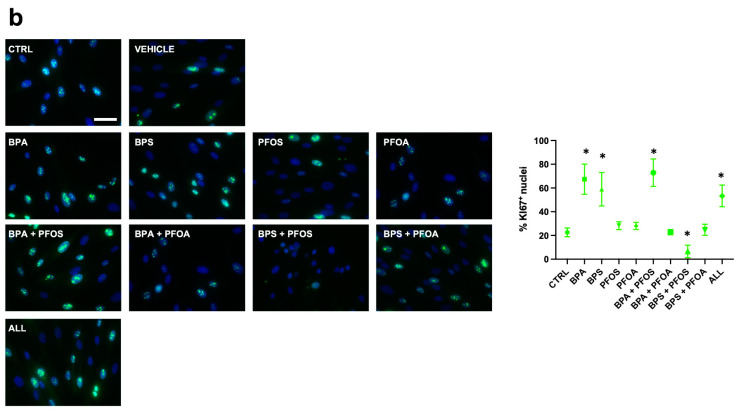
Analysis of PB and PF effects on hFM-MSC proliferation by impedance and Ki67 measurements. On day, 0 hFM-MSCs (displayed in the box, original magnification 20×, and scale bar 50 μm) were treated with 0.1 μM of BPA, BPS, PFOS, or PFOA, alone or in combination, and the impedance values were monitored in real-time up to 48 h. Cells not treated (brown line), treated only with the vehicle (dark blue line), or with lysing agents (purple line) represented the experimental controls. (**a**) Left panel, absolute impedance values (expressed in ohms, Ω). Graph is representative of three different experiments. Right panel, normalized impedance values (Day 0 = 1) of control (brown line) or samples treated with 0.1 μM of BPA, BPS, PFOS, or PFOA alone or in combination, as indicated. Data are expressed as mean ± SD (*n* = 3, * *p* < 0.05 vs. control). (**b**) Immunocytochemical detection of Ki67 (green fluorescence) in hFM-MSC control cells and after 24 h exposure to vehicle or to 0.1 μM of BPA, BPS, PFOS, or PFOA, alone or in combination, as reported. The nuclei were counterstained with DAPI (blue). Original magnification: 40×, scale bar 50 μm. Images are representative of 3 independent experiments. Histogram indicates the % of the positive nuclei in the different experimental conditions. Data are expressed as mean ± SD (*n* = 3, * *p* < 0.05 vs. control). Bisphenol A (BPA), Bisphenol S (BPS), perfluorooctane sulfonate (PFOS), perfluorooctanoic acid (PFOA), BPA + PFOS, BPS + PFOA, BPS + PFOS, BPS + PFOA (ALL).

hFM-MSCs are placenta-derived, fibroblast-like cells obtained from the inner mesodermal tissue of the amniotic membrane (for a review, see [16]) (Figure 1a). These cells were exposed to BPs and PFs for up to 48 h, alone or in combination, and the effects on cell growth and viability were evaluated by impedance analysis. As reported in Figure 1a and Table 1, we found that exposure to BPA, BPS, and PFOS, singularly administered, drives the cells toward proliferation; a similar result was observed in the BPA + PFOS sample and when cells were exposed concomitantly to all the chemicals (ALL), while the combination of BPS + PFOS was highly toxic, inducing a reduction in the impedance value by about 50% relative to control. The effects of 24 h exposure to the xenobiotics on the proliferation of hFM-MSCs were also immunocytochemically evaluated by analyzing the expression of Ki67, a nuclear protein that marks cells in the G2-M phases of the cell cycle (Figure 1b). In accordance with the impedance analysis, we detected a statistically significant increase in Ki67 expression in samples treated with BPA, BPS, and BPA + PFOS and with the combination of all the chemicals, while a reduction in the percentage of Ki67+ nuclei was detected in BPS + PFOS. These results indicate that individually administered BPs interfere with the proliferation of hFM-MSCs; similarly, the combination BPA + BPS and the cocktail of all the EDs drive the cells toward proliferation, while BPS + PFOS appear toxic.

#### 2.1.2. BP and PF Effects on hFM-MSCs Biology: Effects on Mitochondrial Membrane Potential

**Figure 2:**
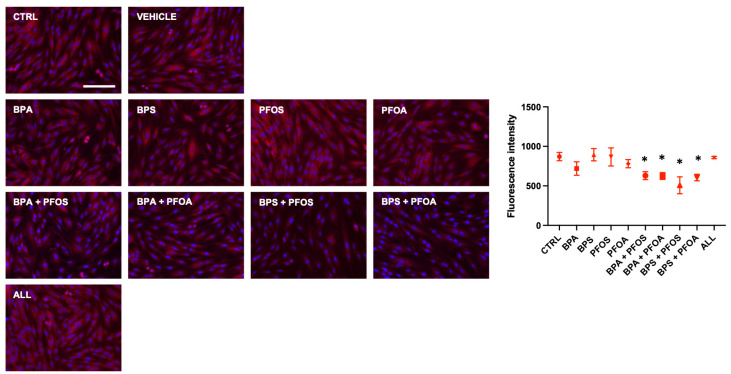
Effects of exposure to BPs and/or PFs on hFM-MSC mitochondrial health. Immunocytochemical detection of MMP (red fluorescence) in control cells and after 24 h exposure to vehicle only or to 0.1 μM of BPA, BPS, PFOS, or PFOA, alone or in combination, as indicated. The nuclei were counterstained with DAPI (blue). Original magnification: 20×, scale bar 100 μm. Images are representative of 3 independent experiments. Histogram indicates the fluorescent intensity (absolute values) in the different experimental conditions. Data are expressed as mean ± SD (*n* = 3, * *p* < 0.05 vs. control). Bisphenol A (BPA), Bisphenol S (BPS), perfluorooctane sulfonate (PFOS), perfluorooctanoic acid (PFOA), BPA + PFOS, BPS + PFOA, BPS + PFOS, BPS + PFOA (ALL).

MitoHealth, a specific red fluorescent probe that accumulates in healthy mitochondria, was used to investigate whether BPs and PFs can harm mitochondrial activity. The results depicted in Figure 2 demonstrate that exposure to the combined (but not to the single) pollutants, for 24 h, affected the mitochondrial membrane potential (MMP), as evidenced by a reduction in staining intensity; unexpectedly, no changes in MMP were observed when samples were concurrently exposed to all the four tested EDs.

#### 2.1.3. Consequences on Stemness Markers Profile

**Figure 3:**
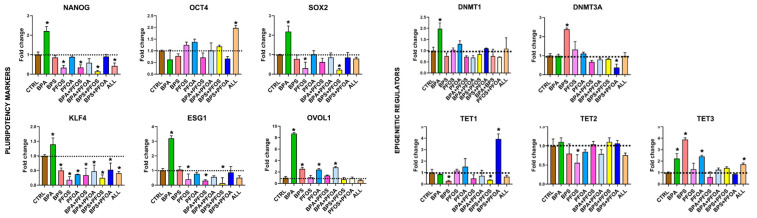
Pluripotency marker and epigenetic regulatory expression in hFM-MSCs upon BPs and/or PFs treatment. The gene expression of the stemness identifiers (NANOG, OCT4, SOX2, KLF4, ESG1, and OVOL1) and epigenetic regulators (DNMT1, DNTM3A, TET1, TET2, and TET3) were detected by qPCR in hFM-MSCs treated with BPs and/or PFs for 24 h, as indicated. The fold changes were determined from the −ΔΔCt values calculated using 18S as a reference gene and normalized to untreated hFM-MSCs as control condition (CTRL). The graphs show the mean ± SD of 3 independent experiments, * *p* < 0.05 vs. CTRL. Bisphenol A (BPA), Bisphenol S (BPS), perfluorooctane sulfonate (PFOS), perfluorooctanoic acid (PFOA), BPA + PFOS, BPS + PFOA, BPS + PFOS, BPS + PFOA (ALL).

Then, it was investigated whether exposure to BPs and PFs affects the pluripotency of the hFM-MSCs. The stemness characteristics of placenta-derived stem cells were evaluated by qPCR analysis of the pluripotency master gene markers. As shown in Figure 3, it was observed that treatment with BPs and PFs, either individually or in combination, affected the stemness of hFM-MSCs to varying degrees. Notably, NANOG expression was upregulated by BPA, while PFOS, alone or in combination with BPA or BPS, determined a dramatic reduction of this transcript; PFOA did not appear to interfere with NANOG expression, while an important decrease was observed when cells were exposed to a cocktail of the four EDs. No OCT4 modifications were detected, except when hFM-MSCs were simultaneously exposed to all four EDs. SOX2 expression was doubled by BPA treatment but dramatically reduced by PFOS, alone or in combination with BPS. Similarly, ESG1 was highly upregulated in hFM-MSCs treated with BPA and strongly curtailed by PFOS, as a single compound or combined with PFs. KLF4 expression was highly downregulated in all experimental conditions, except for the BPA sample, in which a slight increase was detected. Finally, OVOL1, a marker linked to the activation of germinal-specific genes, appeared deregulated by the pollutants, as it was highly (BPA sample) or slightly (BPS, PFOA, and BPA + PFOA samples) upregulated by the treatments.

#### 2.1.4. Modifications of Epigenetic Regulatory Enzymes

Finally, the effect of BP and PF exposure on DNA methyltransferases (DNMTs) and ten-eleven translocation methylcytosine dioxygenases (TETs) involved in epigenetic regulation was investigated, which play a crucial role in the early stages after fertilization [21]. DNMTs are enzymes that add a methyl group to CpG and non-CpG dinucleotide sites. DNMT1 maintains methylation and chromatin stability, while DNMT3A and 3B act as de novo methyltransferases and are crucial for DNA methylation in early embryonic stages [22]. The results indicate that hFM-MSCs express DNMT1 and DNMT3A, but not DNMT3B; the DNMTs were minimally affected by treatments, except for an important DNMT3A reduction observed in the BPS + PFOA sample (Figure 3). On the other side, TETs are responsible for demethylating DNA in regulatory regions and are essential in regulating cell fate during development and in embryonic stem cells by maintaining pluripotency or by regulating differentiation [23]. TET1 was downregulated only in the presence of BPS and BPS + PFOS; TET2 was affected by PFOS, while TET3 expression was upregulated, although to varying degrees, by BPA, BPS, and PFOS and by the cocktail of the four EDs.

### 2.2. BPs and PFs Effects on hAFSCs Biology

#### 2.2.1. Proliferation and Cytotoxicity

**Figure 4:**
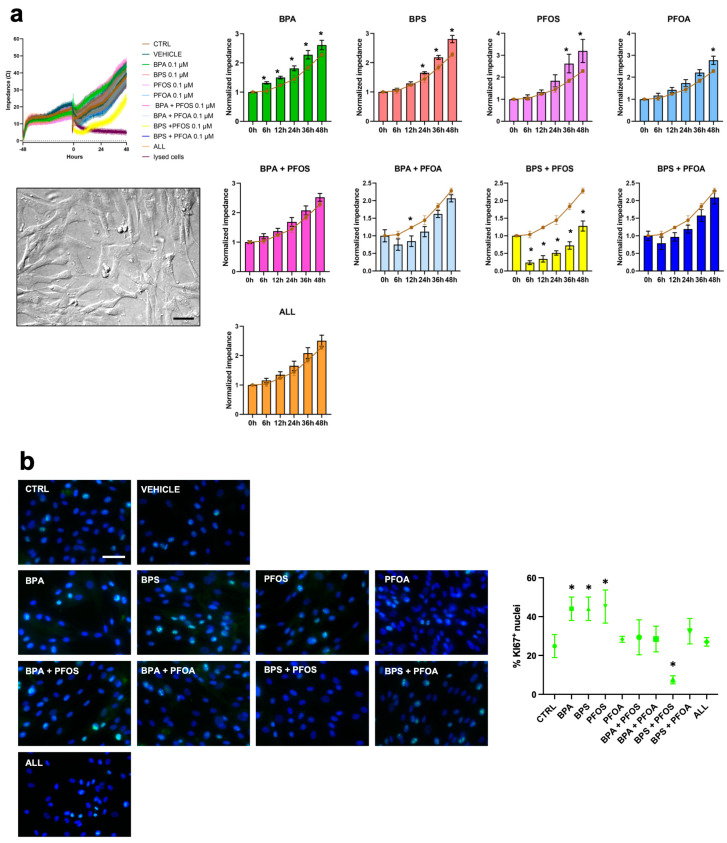
Analysis of PB and PF effects on hAFSC proliferation by impedance and Ki67 measurements. On day 0, hAFSCs (displayed in the box, original magnification 20×, and scale bar 50 μm) were treated with 0.1 μM of BPA, BPS, PFOS, or PFOA, alone or in combination, and the impedance values were monitored in real time up to 48 h. Cells not treated (brown line), treated only with the vehicle (dark blue line), or with lysing agents (purple line) represented the experimental controls. (**a**) Left panel, absolute impedance values (expressed in ohms, Ω). Graph is representative of three different experiments. Right panel, normalized impedance values (Day 0 = 1) of control (brown line) or samples treated with 0.1 μM of BPA, BPS, PFOS, or PFOA, alone or in combination, as indicated. Data are expressed as mean ± SD (*n* = 3, * *p* < 0.05 vs. control). (**b**) Immunocytochemical detection of Ki67 (green fluorescence) in hAFSC control cells and after 24 h exposure to vehicle or to 0.1 μM of BPA, BPS, PFOS, or PFOA, alone or in combination, as reported. The nuclei were counterstained with DAPI (blue). Original magnification: 40×, scale bar 50 μm. Images are representative of 3 independent experiments. Histogram indicates the % of the positive nuclei in the different experimental conditions. Data are expressed as mean ± SD (*n* = 3, * *p* < 0.05 vs. control). Bisphenol A (BPA), Bisphenol S (BPS), perfluorooctane sulfonate (PFOS), perfluorooctanoic acid (PFOA), BPA + PFOS, BPS + PFOA, BPS + PFOS, BPS + PFOA (ALL).

Then, attention was focused on the effects evoked by the xenobiotics on the biological characteristics of the hAFSCs. This cell population is easily accessible with amniocentesis, and it contains fetus-derived stem cells. First, the effects on cell growth and viability were analyzed by impedance analysis. Impedance analysis evidenced that, like the placental cells, the hAFSCs also responded to the exposure that BPA, BPS, and PFOS administered as single compounds, increasing the proliferation rate. No proliferative effects were detected when EDs were paired, except when the combination BPS + PFOS was applied, which determined a dramatic decrease in the cellular impedance to about 50% of the control value (Figure 4a, Table 1). The surge in proliferation due to BPs and PFOS and the toxic effects of BPS + PFOS were then immunocytochemically confirmed by the quantification of the Ki67^+^ nuclei (Figure 4b).

#### 2.2.2. Effects on Mitochondrial Membrane Potential

**Figure 5:**
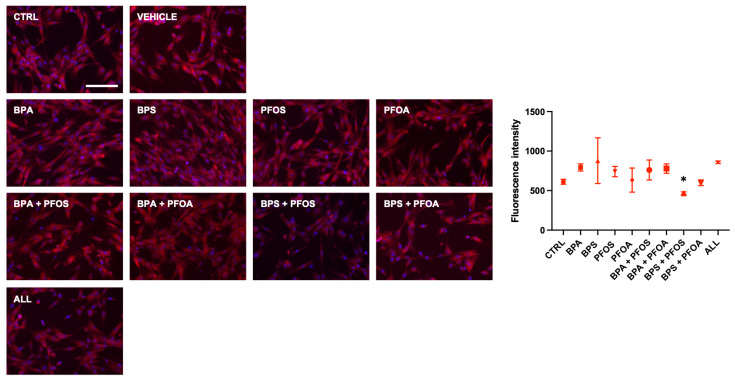
Effects of exposure to BPs and/or PFs on hAFSC mitochondrial health. Immunocytochemical detection of MMP (red fluorescence) in control cells and after 24 h exposure to vehicle only or to 0.1 μM of BPA, BPS, PFOS, or PFOA, alone or in combination, as indicated. The nuclei were counterstained with DAPI (blue). Original magnification: 20×, scale bar 100 μm. Images are representative of 3 independent experiments. Histogram indicates the fluorescent intensity (absolute values) in the different experimental conditions. Data are expressed as mean ± SD (*n* = 3, * *p* < 0.05 vs. control). Bisphenol A (BPA), Bisphenol S (BPS), perfluorooctane sulfonate (PFOS), perfluorooctanoic acid (PFOA), BPA + PFOS, BPS + PFOA, BPS + PFOS, BPS + PFOA (ALL).

Relative to hFM-MSCs, hAFSCs showed a different sensibility to the mitotoxic effects of the xenobiotics, as no modifications in the MMP were detected upon EDs treatment, except for the BPS + PFOS sample, in which an important drop of the MitoHealth staining was shown (Figure 5).

#### 2.2.3. Consequences on Stemness Markers Profile

**Figure 6:**
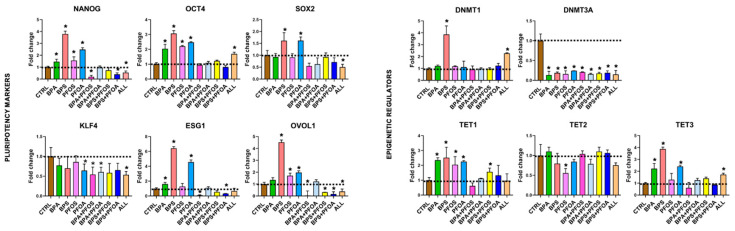
Pluripotency markers and epigenetic regulatory enzymes expression in hAFSCs upon BPs and/or PFs treatment. The gene expression of the stemness identifiers (NANOG, OCT4, SOX2, KLF4, ESG1, and OVOL1), and epigenetic regulators (DNMT1, DNTM3A, TET1, TET2, and TET3) were detected by qPCR in hAFSCs treated with BPs and/or PFs for 24 h, as indicated. The fold changes were determined from the −ΔΔCt values calculated using 18S as a reference gene and normalized to untreated hAFSCs as control condition (CTRL). The graphs show the mean ± SD of 3 independent experiments, * *p* < 0.05 vs. CTRL. Bisphenol A (BPA), Bisphenol S (BPS), perfluorooctane sulfonate (PFOS), perfluorooctanoic acid (PFOA), BPA + PFOS, BPS + PFOA, BPS + PFOS, and BPS + PFOA (ALL).

The impact of BPs and PFs on the expression of pluripotency genes in amniotic stem cells was further examined. All the results relative to gene expression are presented in Figure 6. The master genes of pluripotency were influenced by exposure to BPs and PFs, whether separately or in combination. In particular, hAFSCs increased NANOG expression when treated with the single BPs and PFs, while in the BPA + PFOS and BPS + PFOA samples, a dramatic decrease in the transcript level was detected; a similar result was observed when all the chemicals were administered concomitantly. Unlike hFM-MSCs, OCT4 transcript levels were also enhanced by BPA, BPS, PFOS, and PFOA, singularly applied, and by the cocktail of the four EDs. SOX2 was weakly affected by BPS and PFOA, but its levels were strongly reduced by the combination of all chemicals. As in hFM-MSCs, KLF4 expression tended to decrease in all samples, although a statistically significant difference was observed only when cells were exposed to PFOA, to BPA paired with PFOS or PFOA, or to all the tested EDs concomitantly. ESG1 levels were almost doubled by BPA; BPS and PFOA as single compounds determined an increase of almost 5–6-fold relative to the control, while, on the contrary, their combination curtails ESG1 expression by half with respect to the control; a similar effect was observed in the BPA + PFOS sample. Finally, OVOL1 increased more than 4 folds in the BPS sample, whereas it almost doubled in the PFOS and PFOA samples. When BPS was paired with a PF (PFOS or PFOA) or when all the tested EDs were combined, a dramatic OVOL1 reduction was detected, while in the BPA + PFOS sample, OVOL1 was nearly undetectable.

#### 2.2.4. Modifications of Epigenetic Regulatory Enzymes

Relative to DNMTs, it has been found that hAFSCs also expressed only DNMT1 and DNMT3A and not DNMT3B, but differently from what was observed in the placenta-derived stem cells, EDs treatment affected the expression of the following genes: DNMT1 increased up to 4-fold in the BPA sample and up to 2.5-fold in the ALL sample; conversely, DNMT3A was dramatically reduced in all the experimental conditions (Figure 6). We finally analyzed the effects of EDs on TETs: while TET2 was only scarcely modified by the xenobiotics, TET1 and TET3 were modulated, even if in different ways: TET1 was upregulated by BPs and PFs as a single compound and by BPS + PFOS, while TET3 was increased by BPs and PFOA and by the concomitant exposure to all four pollutants (Figure 6). 

All the data are summarized in Table 2.

## 3. Discussion

This study demonstrated that exposure to BPs and PFs can deeply affect important biological features of hFM-MSCs and hAFSCs, with potential adverse effects on human health. Our main findings can be summarized as follows: (i) Combined exposure to BPS and PFOS proved to be toxic for both hFM-MSCs and hAFSCs; in other tested conditions, BPs and PFs showed minimal or slight effects on the proliferative rate of stem cells. (ii) When combined, BPs and PFs profoundly affected the metabolic status of placental stem cells, modifying the mitochondrial membrane potential (MMP). (iii) BPs and PFs had a profound effect on the biological characteristics of stem cells, including the expression of stemness markers and epigenetic regulators, at least at the transcriptional level, suggesting a dysregulation of the transcription machinery and consequently a potential developmental toxicity. (iv) The effects of a cocktail of different endocrine disruptors (EDs) were not always predictable, as BPs and PFs did not consistently show additive or synergistic effects.

Ethical and methodological issues restrict developmental toxicity studies to animal models, but these may not accurately predict the effects of human antenatal exposure to BPs and PFs due to inter-species hormonal differences. Therefore, in vitro models that mimic the in utero environment are essential for studying physiological processes that are critical for human development, such as the regulation of stem cell self-renewal and differentiation. In our study, we utilized human stem cells derived from both embryos (hAFSCs) and placenta (hFM-MSCs) to investigate the effects of BPs and PFs.

Pregnant women, like all humans, are unintentionally exposed to BPs and PFs. Since the placenta does not represent an effective barrier to these xenobiotics, they can accumulate in the fetus and its annexes, potentially disrupting various metabolic and physiological processes and increasing the risk of developing diseases. Notably, BPA and its alternative BPS have been detected in the human placenta at higher concentrations than in maternal serum, suggesting potential bioaccumulation in perinatal tissues [24,25]. In mice, prenatal exposure to low doses of BPA induces epigenetic alterations and disrupts the sexually dimorphic gene expression patterns of estrogen receptors [26]. Furthermore, PFs have been shown to interfere with thyroid, androgenic, and estrogenic pathways in pregnancy, affecting placental morphology and fetal survival and growth [27,28,29,30]. We already described the sensibility of the diverse human stem cells to different doses of BPs and PFs [31,32]. In this study, the toxic effects of BPs and PFs, alone or in combination, were tested in hAFSCs and hFM-MSCs by evaluating cell proliferation, mitochondrial response, and the expression of genes pivotal for stem cell biology. Unlike many animal studies that employ high doses of EDs, we performed the experiments using BPs and PFs concentrations similar to those found in maternal and infant samples [24,25,28,33,34]. Since humans are exposed to a wide variety of diverse substances simultaneously, the combined effects of these chemicals were examined. The data confirm the challenges in predicting their “cocktail effect”. Indeed, it has been shown that combined exposure to a compound belonging to two different classes does not always result in an additive or synergic effect, but the BPS + PFOS combination appeared to be highly toxic for the stem cells.

To analyze the effects evoked by EDs on stem cell proliferation, we employed MEA biosensor technology on hFM-MSCs and hAFSCs. This approach allowed for a more sensitive and valuable assessment of proliferation through impedance measurements, enabling real-time monitoring without the need for invasive labeling. In addition, it provides constant quality control of the cells, enabling informed decisions about the timing of certain manipulations, such as compound addition. For example, in our case, we added the EDs when cells were in the log phase to test for possible interference with cell growth. Real-time impedance values are more informative than single-time-point values, which can vary dramatically depending on the time the experiment is terminated. The kinetic response analysis revealed that BPs and PFs, administered as single compounds, caused a slight but statistically significant induction of cell proliferation. However, when different ED classes of EDs were combined, no effects were observed, except for the combination of BPS + PFOS, which proved to be toxic for both hFM-MSCs and hAFSCs. Interestingly, exposing hFM-MSCs simultaneously to all xenobiotics led to a surge in proliferation. These observations suggest a complex interaction between BPs and PFs and the cell proliferative machinery. Recent studies demonstrated that PFs disrupt the cell cycle in human breast epithelial cells, while BPs, even at sub-nanomolar concentrations, affect the proliferation of human trophoblast cell lines through ER/MAPK-mediated processes [35,36]. The results from impedance and Ki67 analysis, which is an excellent marker of active cell proliferation since it is expressed in all the cells from the S to M phases of the cell cycle [37], confirm and extend these findings, highlighting that BPs and PFs indeed modify the proliferation of stem cells present in the placenta and amniotic fluid. The tightly regulated proliferative characteristics of stem cells, encompassing self-renewal and differentiation through asymmetric divisions, are particularly crucial during the critical window of development. Any interference in the cell cycle regulation of stem cells during this period may induce permanent alterations in developing tissues and organs.

Exposure to BPs and PFs leads to oxidative stress and disrupts mitochondrial biogenesis, causing a decline in mitochondrial membrane potential (MMP) and inducing mitophagy [38,39]. Mitochondria play a crucial role in cell biology, being responsible for ATP synthesis, the regulation of metabolic pathways, ROS production, and cytosolic Ca^2+^ concentration [40], and the integrity of MMP is vital for preserving mitochondrial structure, function, and metabolism [41]. To assess the effects of BPs and PFs, we employed the MitoHealth assay, a fluorescent live stain that accumulates in mitochondria in proportion to the MMP. Results showed that while the treatments did not impact the mitochondria of amniotic fluid stem cells, the combination of BPs and PFs exhibited statistically significant mitotoxic activity in placenta stem cells. Growing evidence suggests that mitochondrial function and integrity play a crucial role in stem cell viability, proliferation, and differentiation potential [42]. Though the exact molecular mechanisms by which BPs and PFs induce mitotoxicity are not fully elucidated, previous research has indicated that PFs can accumulate inside eukaryotic membranes, altering ion permeability and resulting in changes in membrane potential [43,44].

Pregnancy is a crucial period for studying exposures to EDs due to their potential to impact both the exposed individuals and their offspring. The link between ED exposure and adverse outcomes at birth and later in adulthood [45,46] implies that the fetal epigenome might be a critical target for these substances, affecting developmental processes and disease susceptibility later in life (developmental origins of health and disease). Among the epigenetic mechanisms, DNA methylation regulated by DNMT and TET enzymes plays a vital role in early embryo development by controlling the activation or repression of development-related genes and establishing maternal and paternal imprints. Our findings indicate that while DNMT1, primarily responsible for maintaining methylation during cell replication, was minimally affected by chemical exposure, DNMT3A, essential for de novo methylation, showed diverse effects. BPS + PFOA deeply affected DNMT3A in hFM-MSCs, whereas in hAFSCs, it was statistically significantly downregulated under all tested conditions. TETs were similarly affected by the treatments, with slight alterations observed in hFM-MSCs and more powerful modifications in hAFSCs. These changes in enzyme expression observed in fetus-derived cells could have great consequences. Indeed, DNMT3A shows remarkable fluctuations from oocytes to blastocyst stage and continues to be modified in implanted embryos [22], while TETs control the two demethylation waves characterizing mammalian embryo development [23]. The evidence that BPs and PFs may interfere with epigenetic regulators such as DNMTs and TETs confirms the hypothesis that these EDs can alter fetal reprogramming, thus affecting developmental processes and disease susceptibility later in life.

Finally, this study highlights that these EDs disrupt the network of molecules governing complex early developmental processes, particularly pluripotency markers. In mammalian preimplantation development, proteins like OCT4, NANOG, SOX2, ESG1, and REX1 finely orchestrate pluripotency in stem cells within the inner cell mass, from which somatic and germline cell lineages will derive [47,48]. It was found that BPs and PFs, either alone or in combination, modify the biological characteristics of hFM-MSCs and hAFSCs by altering the expression of pluripotency markers. The finding that BPs and PFs interfere with these genes, which are precisely regulated during pre- and post-implantation phases, further supports the hypothesis of the developmental toxicity of these chemicals. Moreover, in hAFSCs, statistically significant changes were observed in the expression of OVOL1, a gene essential for supporting male gametogenesis. Particularly when EDs were combined, there was a notable reduction in OVOL1 expression. In mice, the removal of Ovol1 results in a dramatic decrease in spermatozoa production as germ cells encounter difficulties progressing through the pachytene stage [49]. The fact that BPs and PFs can downregulate genes critical for spermatogenesis strengthens the association between environmental contaminants and male infertility, suggesting a potential mechanism through which these EDs could impact sperm health.

In conclusion, these findings indicate that even low concentrations of BPs and PFs during antenatal exposure can disrupt the biological characteristics of stem cells, leading to potential defects in human development. These chemicals may also impact the fetal epigenome, which could have implications for health outcomes not only at birth but also later in adulthood. Shedding light on the profound effects of BPs and PFs on stem cell biology, this study underscores the need for further research and awareness regarding the impact of EDs on human health.

### Study Limitations

The study limitations include the use of a single concentration of pooled EDs, which may not fully represent real-life exposure scenarios. Additionally, the study did not explore the molecular mechanism underlying the observed effects and the consequences of longer exposure durations on gene expression and mitochondrial activity. 

## 4. Materials and Methods

### 4.1. Human Cell Cultures and Treatments

Placenta and amniotic fluid samples were obtained after written informed consent in accordance with the Declaration of Helsinki. All samples had normal diploid karyotypes. The study was approved by the ethics committee of St. Orsola-Malpighi University Hospital (2481/2017, ref. 68/2017/U/Tess), and all experiments were performed in accordance with relevant guidelines and regulations. 

hAFSCs were isolated from 3 healthy donor mothers undergoing amniocentesis for prenatal diagnosis at 16–17 weeks of pregnancy, as previously described [50], and cultured in Iscove’s modified Dulbecco’s medium (IMDM) supplemented with 20% fetal bovine serum (FBS), 1% penicillin/streptomycin, 2 mM l-glutamine, and 5 ng/mL basic Fibroblast growth factor (bFGF).

hFM-MSCs were isolated from the term placentas of 3 healthy donor women as previously described [51]. Cells were cultured in DMEM 10% FBS, 1% penicillin/streptomycin, and 2 mM l-glutamine. 

Methanol-dissolved BPA, BPS, PFOS, or PFOA were purchased from Wellington Laboratories (Guelph, ON, Canada) and serially diluted in culture media.

hFM-MSCs and hAFSCs were treated during the log phase with 0.1 μM BPA, BPS, PFOS, and PFOA for 24 h or 48 h. Both cell types were either exposed to a single chemical or to a cocktail in which EDs were paired into the following combinations: BPA + PFOS, BPA + PFOA, BPS + PFOS, BPS + PFOA, and BPA + BPS + PFOS + PFOA (ALL).

### 4.2. Analysis of Impedance

The possible ED cytotoxicity was evaluated by measuring the cell impedance. This method allows real-time monitoring of the cellular response by means of microelectrodes embedded in the culture surface that register cell growth as an increase in impedance, while cell death or detachment corresponds to an impedance decrease. hFM-MSCs and hAFSCs were seeded at 15–20 × 10^5^ on 96-well CytoView MEA plate (Axion Biosystem, Atlanta, GA, USA). During the exponential phase, they were treated with 0.1 μM BPA, BPS, PFOS, or PFOA alone or in combination for 48 h. As previously described [31], impedance values were recorded in real-time by Maestro Edge (Axion Biosystem, Atlanta, GA, USA) using the Impedance Module of Axion Integrated Studio software (Axion Biosystem, Atlanta, GA, USA). The resistance was set at 41.5 kHz.

### 4.3. Immunofluorescence Analysis of Ki67 Expression

hFM-MSCs and hAFSCs were seeded at 15–20 × 10^5^ cell/well in µ-Slide 8 Well (Ibidi, Munich, Germany). During the logarithmic phase, cells were treated with 0.1 μM of BPA, BPS, PFOS, and PFOA, as single compounds or combined, for 24 h. Then, immunofluorescence analysis was performed as previously described [52]. Briefly, cells were fixed in paraformaldehyde 4% and permeabilized by Triton 0.5%. After blocking with BSA 5%, cells were stained with anti-Ki67 antibody 1:100 Alexa fluor 488 conjugated (Thermo Fisher Scientific, Waltham, MA, USA) overnight at 4 °C. Nuclei were counterstained with DAPI 1:1000 (Thermo Fisher Scientific) [53]. Pictures were acquired by EVOS M7000 (Thermo Fisher Scientific) and analyzed by Celleste Image Analysis Software (Thermo Fisher Scientific).

### 4.4. Immunofluorescence Analysis of Mitochondrial Membrane Potential (MMP)

The mitochondrial functionality was analyzed by HCS Mitochondrial Health Kit (Thermo Fisher Scientific), following the manufacturer’s procedures. Briefly, hFM-MSCs and hAFSCs were treated with 0.1 μM of BPA, BPS, PFOS, and PFOA, alone or in combination, for 24 h. Then, cells were incubated with the MitoStain for 30 min at 37 °C and then fixed in paraformaldehyde 4% for 10 min. Nuclei were counterstained by Hoechst 33342. To quantify the fluorescence, 4 different fields for each sample were acquired by EVOS M7000 (Thermo Fisher Scientific) and analyzed by Celleste Image Analysis Software (Thermo Fisher Scientific).

### 4.5. RNA Extraction and Reverse Transcription

hFM-MSCs and hAFSCs, treated with 0.1 μM BPA, BPS, PFOS, and PFOA, individually or in combination for 24 h, were lysed with QIAzol lysis reagent (QIAGEN, Hilden, Germany), and total RNA was extracted using the miRNeasy Mini Kit (QIAGEN, Hilden, Germany), according to the manufacturer’s procedure [54]. For reverse transcription, 1 μg of RNA was retrotranscribed by the High-Capacity cDNA reverse transcription kit (Thermo Fisher Scientific, Waltham, MA, USA), following the manufacturer’s procedure.

### 4.6. Real-Time PCR (qPCR)

For all the examined transcripts, qPCR analysis was performed using SYBR green (PowerUp SYBR Green Master mix, Thermo Fisher Scientific, Waltham, MA, USA) in QuantStudio 3 (Thermo Fisher Scientific, Waltham, MA, USA) [55]. The run method consisted of the following steps: 95 °C for 10 min, 95 °C for 15 s, and 60 °C for 1 min. Steps 2 and 3 were repeated for 40 cycles. The authenticity of the PCR products was verified by melt curve analysis. Each gene expression value was normalized to the 18S ribosomal expression, used as housekeeping. The fold change of each gene was calculated by the ΔΔCT method and expressed in relation to the CTRL. Primer sequences are listed in Table 3.

### 4.7. Statistical Analysis

All data are presented as the mean ± SD of 3 independent experiments. The statistical analysis was performed by Prism 9 (GraphPad, San Diego, CA, USA) using the Student’s *t*-Test. The level of significance was set at *p* < 0.05.

## Figures and Tables

**Table 1:** Effects of a 48 h exposure to the diverse pollutants on cell proliferative rate.

Pollutant	Impedance Value (Fold Change vs. Control)	
	hFM-MSC	hAFSCs
BPA	**1.15**	**1.15**
BPS	**1.27**	**1.23**
PFOS	**1.17**	**1.40**
PFOA	1.11	**1.21**
BPA + PFOS	**1.17**	**0.92**
BPA + PFOA	1.13	1.11
BPS + PFOS	**0.55**	**0.56**
BPS + PFOA	0.94	0.93
ALL	**1.20**	0.92

The bold black and red values represent a statistically significant (*p* < 0.05) increased or decreased foldchange vs. CTRL, respectively.

**Table 2:** The table provides a summary of positive (+) or negative (−) modifications induced by the EDs, as single compound or combined, proliferation rate, mitochondrial health, and gene expression in perinatal stem cells.

hFM-MSCs			**BPA**	**BPS**	**PFOS**	**PFOA**	**BPA + PFOS**	**BPA + PFOA**	**BPS + PFOS**	**BPS + PFOA**	**ALL**
	Proliferation Rate	Impedance	+	+	+		+		−		+
		Ki67^+^	++	+			++		−−		+
	Mitochondrial Health	MMP					−	−	−	−	
	Pluripotency Markers	*NANOG*	+		−−		−−		−−		−−
		*OCT4*									+
		*SOX2*	+		−−				−−		
		*ESG1*	++		−−		−−		−−		
		*KLF4*	+	−	−−	−	−−	−	−−	−	−−
		*OVOL1*	+++	+		+		+			
	Epigenetic Regulators	*DNMT1*	+								
		*DNMT3A*	+							−−	
		*TET1*		−−					−−	++	
		*TET2*			−						
		*TET3*	+	++	+						+
hAFSCs	Proliferation Rate	Impedance	+	+	+	+			−		
		Ki67^+^	+	+	+	+			−−		
	Mitochondrial Health	MMP							−		
	Pluripotency Markers	*NANOG*	+	++	+	+	−−			−−	−
		*OCT4*	+	++	+	+					+
		*SOX2*		+		+					−
		*ESG1*	+	+++		+++	−−			−−	
		*KLF4*				−	−	−			−
		*REX1*									
		*OVOL1*		++	+	+	−−		−−	−−	−−
	Epigenetic Regulators	*DNMT1*		++							+
		*DNMT3A*	−−	−−	−−	−−	−−	−−	−−	−−	−−
		*TET1*	+	+	+	+			+		
		*TET2*			−						
		*TET3*	+	++		+					+

MMP: Mitochondria Membrane Potential. The entity of the modification was considered, using three categories for increasing values (+ = up to 3-fold increase, ++ = between 3- and 5-fold increase, and +++ = more than 5-fold increase), and two categories for decreasing values (− = up to 0.5-fold decrease, − − = more than 0.5-fold decrease).

**Table 3:** This table includes the primer sequence for qPCR.

Gene	Sequence (5′ to 3′)	Reference
*NANOG_FW*	CCAGACCCAGAACATCCAGTC	[17]
*NANOG_REV*	CACTGGCAGGAGAATTTGGC	
*OCT4_FW*	GGGTTTTTGGGATTAAGTTCTTC	[56]
*OCT4_REV*	GCCCCCACCCTTTGTGTT	
*SOX2_FW*	CAAAAATGGCCATGCAGGTT	[56]
*SOX2_REV*	AGTTGGGATCGAACAAAAGCTATT	
*ESG1_FW*	CCATGAATGCCCTCGAACTAGG	[19]
*ESG1_REV*	CCTTAACTCTTTAGGCTGGAGCA	
*KLF4_FW*	AGCCTAAATGATGGTGCTTGGT	[56]
*KLF4_REV*	TTGAAAACTTTGGCTTCCTTGTT	
*OVOL1_FW*	AGAGCAGAGACCATGGCTTC	[50]
*OVOL1_REV*	GACGTGTCTCTTGAGGTCGA	
*DNMT1_FW*	GCCAGAGATAGAGATCAAGCTG	Primer blast
*DNMT1_REV*	CACAGCGTGTCAGAGATGCC	
*DNTM3A_FW*	CCATCGTCAACCCTGCTCG	Primer blast
*DNMT3A_REV*	CACCACATTCTCAAAGAGCCAG	
*DNTM3B_FW*	GACTCGTTCAGAAAGCCCAG	Primer blast
*DNMT3B_REV*	GGACTCGTCCACATGGTTGC	
*TET1_FW*	ACTCCCTGAGGTCTGTCCTG	Primer blast
*TET1_REV*	CAGGTAGGGCTGCATGACTT	
*TET2_FW*	CTCAGCAGCAGCCAATAGGA	Primer blast
*TET2_REV*	CTGTCTGGCAAATGGGAGGT	
*TET3_FW*	AACTGCTCACTCAGCTCTGC	Primer blast
*TET3_REV*	GCAGCCCTCAGAAAAGGGAT	
*18S_FW*	CATGGCCGTTCTTAGTTGGT	[57]
*18S_REV*	CGCTGAGCCAGTCAGTGTAG	

## Data Availability

Data are available from the authors on reasonable request.
